# Statistical Recommendations for Immunity, Inflammation and Disease

**DOI:** 10.1002/iid3.70466

**Published:** 2026-06-11

**Authors:** Michal Ordak

**Affiliations:** ^1^ Centre of Regenerative Medicine Medical University of Bialystok Bialystok Poland

**Keywords:** immunity, inflammation, statistical recommendations

*Immunity, Inflammation and Disease* is an interdisciplinary, peer‐reviewed, open‐access journal that publishes research spanning the full spectrum of immunology. It considers both fundamental and clinical studies, aiming to support the timely dissemination of findings across diverse areas of immune‐related science. The journal is widely indexed and welcomes contributions that advance understanding of immune mechanisms, diseases, and translational applications. The aim of this brief article is to outline key statistical recommendations for authors planning to submit manuscripts to the journal.

The World Association of Medical Editors recommends the SAMPL (Statistical Analyses and Methods in the Published Literature) guidelines, which constitute a set of fundamental principles for reporting statistical methods in biomedical research [1]. They are designed to help authors and reviewers ensure that statistical analyses are described clearly, accurately, and transparently, and are also supported by the EQUATOR Network to promote high standards in research reporting. As a first recommendation, authors submitting manuscripts to Immunity, Inflammation and Disease should familiarize themselves with the SAMPL guidelines prior to submission. Early adherence to these principles has been shown to improve the clarity and transparency of statistical reporting, potentially reducing the need for extensive revisions and lowering the risk of avoidable rejection [2].

As a second recommendation, authors may benefit from consulting a review published in August 2025 in Allergy, the official journal of the European Academy of Allergy and Clinical Immunology (EAACI), a leading journal in the field of allergy and clinical immunology. The article, “Statistical Analysis in Allergy and Immunology: A Review With Practical Examples”, provides a comprehensive and practically oriented overview of key statistical principles, including transparent reporting of methods, alignment of statistical analyses with study design and research questions, assessment and handling of model assumptions, appropriate selection of statistical tests depending on data characteristics, as well as the interpretation of statistical significance alongside clinical relevance [3]. Given that *Allergy* represents a closely related field encompassing both allergy and immunology, the practical focus of this review is particularly relevant to authors submitting to Immunity, Inflammation and Disease. The emphasis on real‐world examples and well‐justified analytical approaches highlights good statistical practice in applied biomedical research. This practical perspective may support authors in translating general statistical principles into appropriately designed and clearly reported analyses.

The third recommendation is that authors should clearly indicate in the cover letter which co‐authors were responsible for conducting the statistical analysis. This information enhances transparency and allows editors and reviewers to better assess the methodological credibility of the study. It also helps clarify the distribution of responsibilities within the research team, particularly in relation to data handling and interpretation. When the analysis has been performed by a qualified biostatistician, this should be explicitly acknowledged. Such disclosure may increase confidence in the robustness and reliability of the reported results.

Lastly, authors should ensure that the data underlying the analyses are made publicly accessible whenever possible. Providing open access to datasets facilitates independent verification of the results and strengthens the reproducibility of the study. It also allows other researchers to explore the data without the need for individual data requests. Such practices contribute to greater transparency and trust in the reported findings.

In conclusion, the statistical recommendations outlined for *Immunity, Inflammation and Disease* aim to support authors in preparing high‐quality and methodologically sound manuscripts. Adherence to clear and transparent reporting principles is essential for ensuring the credibility and reproducibility of biomedical research. The integration of established guidelines and good reporting practices can improve the consistency and interpretability of submitted work. These recommendations may also facilitate the peer review process and reduce the likelihood of avoidable revisions or rejection. Ultimately, strengthening statistical rigor is essential for advancing reliable scientific knowledge in immunology and related fields.

## Author Contributions

The author contributed to all aspects of the manuscript.

## Ethics Statement

The author has nothing to disclose.

## Conflicts of Interest

The author declares no conflicts of interest.

## Data Availability

Data sharing not applicable to this article as no datasets were generated or analysed during the current study.
